# Systems biology of platelet-vessel wall interactions

**DOI:** 10.3389/fphys.2013.00229

**Published:** 2013-08-26

**Authors:** Scott L. Diamond, Jeremy Purvis, Manash Chatterjee, Matthew H. Flamm

**Affiliations:** Department of Chemical and Biomolecular Engineering, Institute for Medicine and Engineering, University of PennsylvaniaPhiladelphia, PA, USA

**Keywords:** platelet, thrombosis, hemodynamic, ADP, thromboxane

## Abstract

Blood systems biology seeks to quantify outside-in signaling as platelets respond to numerous external stimuli, typically under flow conditions. Platelets can activate via GPVI collagen receptor and numerous G-protein coupled receptors (GPCRs) responsive to ADP, thromboxane, thrombin, and prostacyclin. A bottom-up ODE approach allowed prediction of platelet calcium and phosphoinositides following P2Y_1_ activation with ADP, either for a population average or single cell stochastic behavior. The *homeostasis assumption* (i.e., a resting platelet stays resting until activated) was particularly useful in finding global steady states for these large metabolic networks. Alternatively, a top-down approach involving *Pairwise Agonist Scanning* (PAS) allowed large data sets of measured calcium mobilization to predict an individual's platelet responses. The data was used to train neural network (NN) models of signaling to predict patient-specific responses to combinatorial stimulation. A kinetic description of platelet signaling then allows prediction of inside-out activation of platelets as they experience the complex biochemical milieu at the site of thrombosis. Multiscale lattice kinetic Monte Carlo (LKMC) utilizes these detailed descriptions of platelet signaling under flow conditions where released soluble species are solved by finite element method and the flow field around the growing thrombus is updated using computational fluid dynamics or lattice Boltzmann method. Since hemodynamic effects are included in a multiscale approach, thrombosis can then be predicted under arterial and venous thrombotic conditions for various anatomical geometries. Such systems biology approaches accommodate the effect of anti-platelet pharmacological intervention where COX1 pathways or ADP signaling are modulated in a patient-specific manner.

## Introduction

A systems biology approach for platelets seeks to predict kinetic processes during clotting or bleeding episodes. A multiscale modeling framework should facilitate the bridging of genomics/proteomics studies with platelet phenotype and vessel pathophysiology under hemodynamic conditions. Such a framework should quantify risks and severity of such episodes for a given phenotype/genotype as well as the safety and efficacy of pharmacological intervention.

### Platelet genomics

Genome-wide association studies (GWAS) have found rather modest associations of SNPs (single nucleotide polymorphisms) with thrombosis or vascular disease (Wellcome Trust Case Control Consortium, 2007; Ouwehand, 2007). A GWAS focused specifically on coronary artery disease (CAD) identified 4 loci linked to CAD (Samani et al., 2007), but mechanistic understanding of these SNPs awaits exploration and may not necessarily be platelet-linked. Jones et al. (2007) measured platelet response, calcium mobilization, aggregometry, and FACS determination of response to ADP or GPVI activation with collagen-related peptide (CRP) in 506 healthy volunteers to define inter-individual variability. Importantly, SNPs in the GP6 locus were linked to about 35% of the variation in response to CRP. Variability in response to ADP was associated with polymorphisms in the platelet P2Y_12_ receptor (Fontana et al., 2003). However, it currently remains difficult to use GWAS or genotyping information to predict risk for a specific patient. Greliche et al. (2013) conclude from their genome-wide SNP interaction analysis that common SNPs were unlikely to have strong interactive impact on the risk of venous thrombosis. Few if any genomic studies quantitatively predict an individual's blood function during clotting or bleeding disease scenarios under hemodynamic conditions.

### Platelet transcriptome and proteomics

Platelets contain a microRNA (miR) pool and a translatable mRNA pool that declines with a platelet's age in the circulation. McRedmond et al. (2004) identified 2928 mRNA species using Affymetrix arrays. Many of the top 50 most abundant platelet mRNAs correlated with secreted or membrane proteins such as β_2_-microglobulin, platelet factor 4, factor XIII, GPIb, α_IIb_, etc. Similarly, Gnatenko et al. (2003) found 2147 mRNA species and Bugert et al. (2003) found 1526 mRNA species in purified platelets. More recent direct sequencing methods identified ~9000 mRNA species in platelets (Bray et al., 2010), more comparable in size to the transcriptome of megakaryocytes. Also, platelets contain a functional spliceosome. For example, the processing of tissue factor (TF) mRNA and translation of TF upon platelet activation was found in activated platelets (Schwertz et al., 2006), although TF activity was only detectable in sonicated platelet membranes. The human platelet proteome has been examined and an important web-based resource is now available to explore protein-protein interactions within platelets (Boyanova et al., 2011).

Inter-individual variation in platelet reactivity, even in a normal population, has been associated with a number of factors including: female gender, fibrinogen level, ethnicity, inherited variations, and polymorphisms (Hetherington et al., 2005; Bray, 2007). Unfortunately, no single genomic or proteomic factor is a strong predictor of hyper-reactivity in typical subjects and the need for advanced functional phenotyping motivates the development of systems biology tools to quantify blood function.

### Clotting under flow conditions

Collagen is sufficient to capture and activate platelets under venous wall shear rates (γ_w_ ~100–200 s^−1^). In the arterial circulation (γ_w_ ~1000–2000 s^−1^), collagen adsorbed von Willebrand factor (vWF) facilitates platelet capture, allowing collagen induced GPVI signaling and subsequent α_2_β_1_ and α_2b_β_3_ activation. Under flow conditions, red blood cells help enrich the platelet concentration by ~3–8× in the plasma layer near the wall. At pathological high shear exposures (>5000 s^−1^) encountered in severe stenosis, mechanical heart valves, and continuous LVAD pumps, the plasma vWF may undergo structural changes such as a transition from a globular to an extended state (Schneider et al., 2007), likely increasing the availability of A1 domains in the vWF polymer for multivalent contacting with platelet GPIb receptors. Interestingly, severe stenosis and LVAD pumps can lead to an acquired von Willebrand disease, demonstrating the importance of local hemodynamics on the systemic circulation.

### Growth of the platelet aggregate via autocatalytic signaling

Collagen triggers GPVI clustering, leading to rapid phosphorylation of the GPVI-associated Fc receptor by Src family tyrosine kinases. Such phosphotyrosine residues are recognized by Syk, and the binding and activation of Syk activates PLC_γ2_. PLC_γ2_ converts phosphatidylinositol (PI)-4,5-P_2_ (PIP_2_) to inositol 1,4,5-trisphosphate (1,4,5-IP_3_ or IP_3_) and diacyclglycerol (DAG). IP_3_ opens Ca^2+^ channels in the platelet dense tubular system (DTS). Depletion of DTS Ca^2+^ results in STIM1 activation and binding to Orai1, leading to store operated calcium entry (SOCE). DAG/Ca^2+^ activates protein kinase C (PKC) in platelets, which in turn governs several serine/threonine phosphorylation events.

Beyond the first monolayer of platelets adherent to collagen/VWF, the addition of subsequent layers of platelets to the growing thrombus is strongly potentiated by locally released ADP and thromboxane (TXA_2_) as well as locally generated thrombin. ADP activates P2Y_1_ and P2Y_12_ while TXA_2_ activates the TP receptor and thrombin cleaves PAR1 and PAR4. Activation of a GPCR causes an exchange of GTP for GDP on the α subunit of the G protein and dissociation of the α and β γ subunits. Both these units in turn interact with secondary effectors such as PLCβ and adenylate cyclase. Human platelets express at least 10 forms of Gα (including members of the Gq, Gi, G12, and Gs families) (Brass et al., 2006; Offermanns, 2006). Thrombin, ADP, and TXA_2_ activate PLCβ via Gq. PLCβ generates IP_3_ from membrane PIP2. Rising Ca^2+^ levels activate the Ras family member, Rap1B via Cal-DAG GEF. Rap1B activation is a precursor to α_IIb_β_3_ activation and allows the platelets to form aggregates with other platelets through fibrinogen cross-bridging. Ca^2+^-dependent signaling drives myosin light chain kinase and activation of GTP binding proteins of the Rho family (Klages et al., 1999). Rho activation in turn activates kinases like p160ROCK and 5 LIM-kinase that can phosphorylate myosin light chain kinase and cofilin to regulate actin-dependent cytoskeletal shape changes (Pandey et al., 2006).

Endothelial derived prostacyclin (PGI_2_) binds the IP receptor and causes Gs mediated increase in adenyl cyclase activity. Also, NO from the endothelium and platelets can activate guanylate cyclase resulting in elevated cGMP levels that subsequently inhibit the hydrolysis of cAMP by intracellular phosphodiesterases. Taken together these mechanisms elevate intracellular cAMP levels, which strongly downregulate platelet signaling. Agonists coupled to Gi family members inhibit cAMP production in platelets, thus allowing activation to proceed unhindered (Yang et al., 2002). Additionally the βγ subunits of these receptors can activate PLCβ and the γ isoform of PI3K. The effectors for PI3K include Rap1b and Akt (Woulfe et al., 2002).

ADP is stored in platelet dense granules and is released upon activation. P2Y_1_ and P2Y_12_ are the primary receptors for this agonist. P2Y_1_ is Gq coupled and signaling through this receptor causes Ca^2+^ mobilization, shape change, and thromboxane generation. P2Y_12_ is the target of the commonly used anti-platelet drug Plavix, and is a Gi2 coupled receptor that inhibits cAMP production in platelets.

Thrombin is a potent platelet agonist that causes fast mobilization of intracellular Ca^2+^, and activation of phospholipase A_2_ and subsequent thromboxane generation (Offermanns et al., 1997). Also, thrombin can trigger Rho dependent signaling pathways in platelets (Moers et al., 2003), that contribute to actin modeling and shape change. Thrombin signals through the protease-activated receptor (PAR) family of GPCRs. PAR1 and PAR4 are expressed on human platelets, while PAR3 and PAR4 are expressed on mouse platelets. Thrombin cleaves the N-terminus of these receptors, exposing a new N-terminus that serves as a tethered ligand for these receptors. Synthetic peptides are able to selectively activate these receptors and mimic the actions of thrombin (for example, SFLLRN for PAR1, and AYPGKF for PAR4). Kinetic studies have shown that the human platelet response to thrombin is biphasic and involves first signaling through PAR1 and subsequent signaling through PAR4 (Covic et al., 2000). In mouse platelets signaling occurs primarily via PAR4, and is facilitated by PAR3. In addition to the PAR receptors, GP1bα has high affinity for thrombin. Absence of GP1bα reduces responses to low doses of thrombin and diminishes PAR1 signaling, suggesting that this receptor facilitates signaling through the PARs (Dormann et al., 2000). Ca^2+^ mobilization also activates phospholipase A_2_ (PLA_2_), which in turn converts membrane phospholipids to arachidonic Acid. TXA_2_ is produced from membrane arachidonate by the aspirin sensitive cyclooxygenase (COX-1) enzyme. TXA_2_ causes Ca^2+^ mobilization, aggregation, secretion, phosphoinositide hydrolysis, and protein phosphorylation. TXA2 can diffuse across the membrane and activate nearby platelets, but its activity is limited by the molecule's short half life (~30 s).

## Systems biology models of platelet-vessel wall interactions

### Bottom-up model of ADP activation of P2Y_1_ receptor

**Figure 1:**
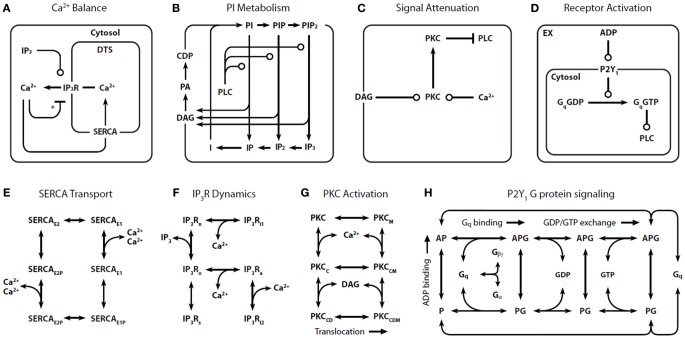
**Detailed reaction schemes for platelet signaling modules.** Four interconnected models were defined: **(A)** Ca^2+^ module: cytosolic and DTS compartments are separated by the DTS membrane, which contains the IP3R and SERCA. **(B)** Phosphoinositide (PI) module: Membrane-bound PIs are cleaved by PLC-β to form diffusible inositol phosphates and DAG, which are substrates for resynthesis of PIs. **(C)** PKC module: Ca^2+^_*i*_ and DAG activate PKC, which migrates to the plasmamembrane where it phosphorylates PLC-β. **(D)** P2Y_1_ module: extracellular ADP binds to and activates P2Y_1_. Active P2Y_1_ accelerates guanine nucleotide exchange on bound Gq. The Gq·GTP binds and activates PLC-β, which increases the GTPase activity of Gq·GTP. Molecular kinetic descriptions are embedded in the signaling modules for: **(E)** SERCA catalytic cycle (Dode et al., 2002): Subscripts: E1, facing cytosol; E2, facing DTS; P, phosphorylated. **(F)** IP3R dynamics (Sneyd and Dufour, 2002): Subscripts: n, native; i1, inhibited; o, open; a, active; s, shut, i2, inhibited. **(G)** PKC activation: Active kinase is bound to Ca^2+^_*i*_ and DAG and located at the PM. Subscripts: M, located at the PM; C, Ca^2+^-bound; D, DAG-bound. **(H)** P2Y_1_ activation module: Rate equations describing the interactions among ADP, P2Y1, and Gq were modeled according to the ternary complex model described in Kinzer-Ursem and Linderman (2007). Abbreviations: A, ADP; P, P2Y1; G, Gq. ^*^Ca^2+^ both activates and inhibits IP3R.

In our representation of P2Y_1_ activation (Figure 1) (Purvis et al., 2008), binding of extracellular ADP to P2Y1 leads to activation of Gq through GDP/GTP exchange reactions. Gq·GTP is a substrate for GTPase activating proteins (GAPs) such as PLC-β and RGS4, which can accelerate Gq·GTP hydrolysis over 1000-fold. Although other ADP receptors are involved in platelet Ca^2+^ signaling (e.g., P2X_1_ and P2Y_12_), the P2Y_1_ receptor (~150 copies/platelet) contributes 90% of the Ca^2+^ signal.

**Figure 2:**
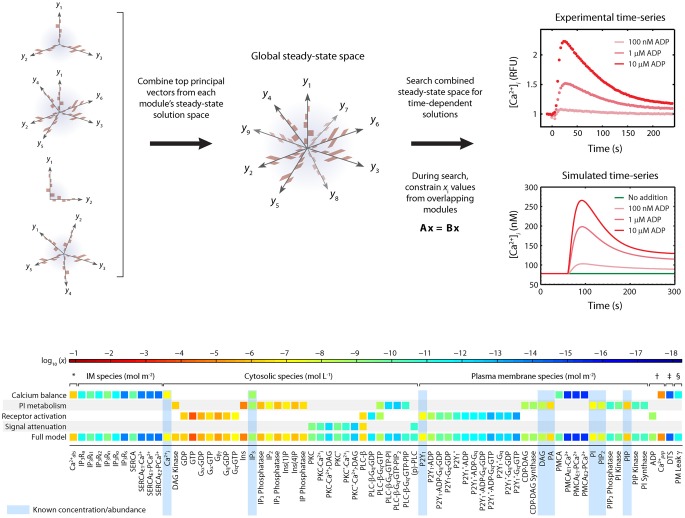
**Homeostasis requirement: Assembly of full model from steady-state modules using principle component analysis (PCA).** The full model is assembled by combining PCA-reduced, steady-state solution spaces from each module into a combined steady state solution space. This global space is searched for full-length, steady-state solution vectors that satisfy both the steady state requirements of each module and the desired time-dependent properties when the steady-state is perturbed. A simple linear constraint is imposed for every pair of modules that share a common molecule *c*_*i*_ to ensure that steady state solutions are consistent. To assemble the platelet signaling model, a set of 16 PC vectors representing all 72 unknown variables in the model were used as search directions in a global optimization routine. The global solution space was searched for models with accurate dynamic behavior using experimental time-series data for ADP-stimulated Ca^2+^ release. Species are grouped according to compartment. Color values correspond to molar concentrations (mol/L or mol/m^2^) or as indicated: ^*^DTS species (mol L^−1^). ^†^Extracellular species (mol L^−1^). ^‡^DTS volume (L). ^§^PM leak conductance/area (S m^−2^).

**Figure 3:**
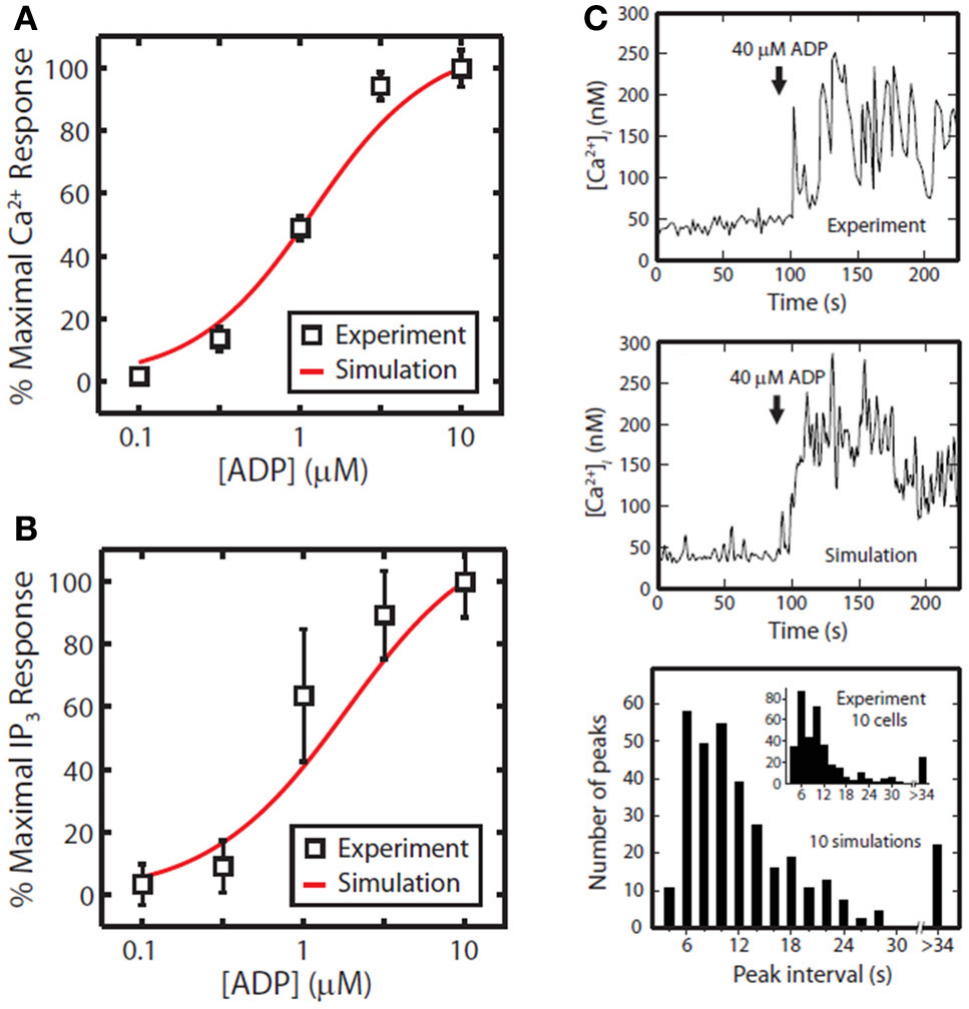
**Tests of P2Y1 signaling model.** ADP dose response for the full platelet model from 100 nM to 10 μ M ADP for calcium mobilization **(A)** or IP3 generation **(B)**. Stochastic simulation of a single platelet **(C)**. A single, fura-2-loaded platelet was immobilized on a fibrinogen-coated coverslip and activated with 40 μM ADP at *t* = 90 [Ca^2+^ trace from Heemskerk et al. (2001)]. After 90 s of simulated rest, the platelet model was activated by setting extracellular [ADP] to 40 μ M. Simulated interval times were binned in 2 s increments for direct comparison with experiment (*inset*).

**Figure 4:**
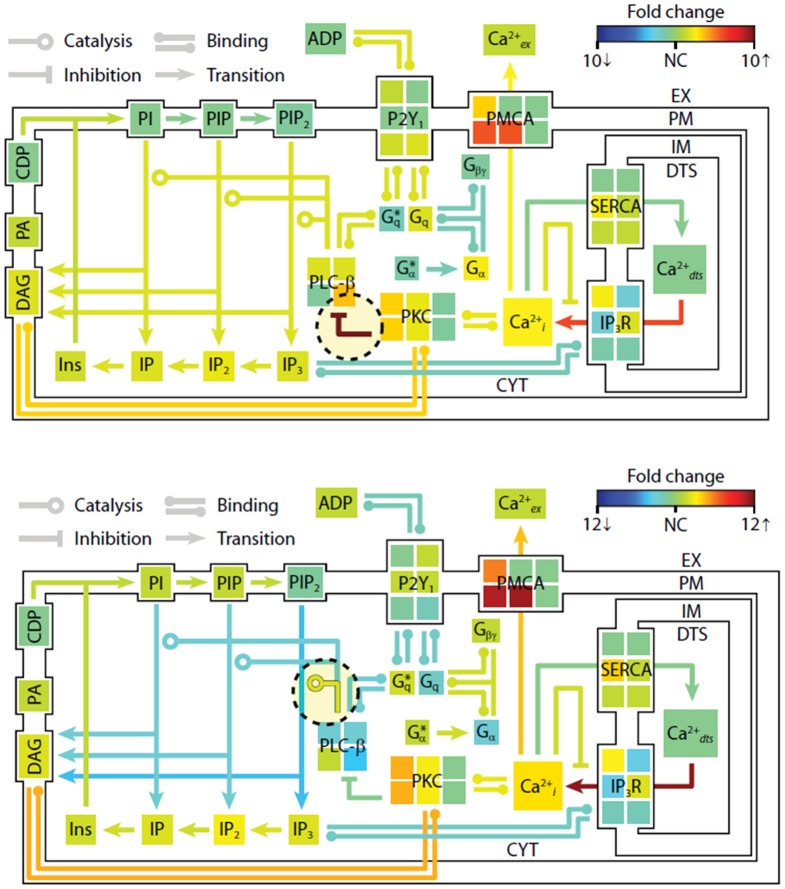
**Shifts in steady-state profiles caused by kinetic perturbations.** The steady-state platelet model was perturbed by changing selected kinetic parameters (*±*10-fold) and simulating for 1 h. After approaching a new steady state, the model concentrations and fluxes were determined relative to their original steady-state values and colored according to fold-change. Green indicates no change (NC) relative to initial flux/concentration. Red indicates a relative increase and blue indicates a relative decrease. Note that the color scale in each panel is normalized separately to maximize distinctions in fold change. New steady states were achieved after (**top**) 10-fold decrease in PKC-mediated inhibition of PLC-β, and (**bottom**) 10-fold increase in PIP2 hydrolysis (10-fold increase in *kcat* of hydrolysis). ^*^, active state.

Because of the inherent complexity in a model of this size, we constructed four signaling “modules”: (1) Ca^2+^ release and uptake (Figure 1A), (2) phosphoinositide (PI) metabolism (Figure 1B), (3) PKC regulation of phospholipase C-β (PLC-β) (Figure 1C), and (4) P2Y1 G-protein signaling (Figure 1D). These modules use previously validated or data-consistent kinetic networks for SERCA, IP3-Receptor, PKC translocation, and GPCR signaling (Figures 1E–H).

Assembling the four modules together results in a global ODE model that has 77 reactions, 132 fixed kinetic rate constants, and 70 species. Since the reaction network (Figure 1) and the kinetic parameters are fixed, the reaction topology of the model is also fixed. Such a model takes the general form: *d****c***/*dt* = *F(**c***) and ***c***(*t = 0*) = ***c***_*o*_ where ***c*** is a vector of all species concentrations and ***c***_*o*_ is a specified initial condition vector at *t* = 0.

To determine appropriate sets of ***c***_*o*_ that are suitable for use in modeling platelets, a challenge exists that the copy number of each species in a resting platelet is not known. Imposing a *homeostasis assumption* results in powerful tool to define a set of acceptable ***c***_*o*_ vectors. The homeostasis assumption states that a resting platelet remains resting until activated. This means that an acceptable initial condition ***c***_*o*_ also represents a steady state for the system and will satisfy the equation *d****c****/dt* = 0. Finding a global ***c***_*o*_ involves assembling the steady state solutions of each module (Figure 2).

The first phase of the method involves generating a compact representation of the steady-state solutions for each module. First, conservative bounds are chosen for ***c*** based on physiological and practical considerations. Also, because molecular concentrations can span several orders of magnitude, it is most efficient to delineate this range of values on a logarithmic scale rather than a linear scale. Once the sampling distribution for **c** has been defined, steady-state solutions (***c***_*o*_ = ***c*_*ss*_**) for each module are calculated using fixed kinetic parameters for each reaction in the module. For non-oscillating systems, steady-state solutions may be obtained by simulating the system until equilibrium is reached (i.e., until *d****c****/dt* = 0). In the third step, a large collection of steady-state solutions for each module is subjected to principal component analysis (PCA) (Purvis et al., 2009). PCA is then used to transform these points to a new coordinate set that optimally covers the space of steady-state solutions using the fewest number of dimensions. For example, if two molecule concentrations in the steady-state space are highly correlated due to participation in the same reaction, PCA will locate a single dimension to represent each pair of points in the transformed space. Ultimately, these new dimensions will be combined across all modules to search for global solutions that lie in the steady-state space for the fully combined network. Since PCA is a linear method, a steady-state solution space that is highly nonlinear may require more principal component vectors to accurately estimate the solutions. The reduction procedure is shown for the human platelet model comprising 4 interlinked signaling modules (Figure 2). For this step, we generated more than 10^9^ sets of initial guesses (***c***_*o*_) for each module, computed the initial value problem for each ***c***_*o*_ until a steady state was reached (*d****c****/dt* ≈ 0), and selected only those steady states (***c*_ss_**) that were consistent with known concentrations (i.e., [Ca^2+^]_*o*_ ~100 nM). Interestingly, only a small fraction of initial guesses produce steady-state solutions that are also consistent with known concentration values. For example, it was shown that only 50,000 of 10^9^ initial guesses (0.005%) in the Ca^2+^ balance module (Figure 1A) met both requirements and were suitable for further analysis. This observation shows that the kinetic topology of these molecular networks places very strong constraints on the range of concentrations that can exist at steady state. In biological terms, this suggests that fixed kinetic properties at the molecular level (e.g., IP3R and SERCA kinetics) can affect not only the dynamical features of a biochemical system but can also determine the abundance of chemical species and the compartmental structures that contain them. A fully assembled initial condition vector results (bottom, Figure 2) results in new hypotheses about allowable concentrations and ratios of concentrations (i.e., IP3/SERCA ratio is very small). The allowed ***c**_*o*_* = ***c**_ss_* is consistent with the known resting levels of Ca2+, IP_3_, P2Y1, DAG, PA, PI, PIP_2_, and PIP (bottom, Figure 2) as well as the stimulated response of platelets to increasing amounts of ADP (right, Figure 2). With a global simulation of P2Y1 signaling, it is possible to simulate the ADP dose-response of calcium mobilization and IP3 generation in platelets as well as the mobilization of intracellular calcium in a single platelet due to stochastic fluctuations (Figure 3).

Since many initial condition vectors can be found to allow a resting platelet to remain resting and then respond appropriately to stimulation, investigation of these multiple steady states and associated cell responses can allow an *ad-hoc* sensitivity analysis. Some species (flexible nodes) may vary widely in the allowed initial condition vectors but have little effect on system response. In contrast, other species (rigid nodes) may be forced to take on values in a very narrow range due to the kinetic constraints of the problem.

To examine the changes in steady-state properties caused by kinetic perturbations in the P2Y1 model, we altered the rates of important regulatory reactions and observed the system response to each perturbation. Each perturbation cause a brief adjustment phase lasting ~200 s followed by a more gradual phase characterized by a new steady-state profile. After 1 h of simulated time, steady-state concentrations and reaction fluxes were quantified relative to their original steady-state levels (Figure 4). In a computational perturbation, the inhibition of phospholipase C-β (PLC-β) activity by PKC was reduced 10-fold. Since PKC has a negative-feedback role in suppressing the platelet-stimulating activity of PLC-β, this perturbation caused a 2-fold increase in steady-state PIP_2_ hydrolysis, elevated IP_3_ concentration, and accelerated Ca^2+^ release. This was a compensatory effect caused by the negative feedback loop involving Ca^2+^-regulated activity of PKC, a resulting new hypothesis that can be probed experimentally. In another example, increasing the hydrolytic activity of PLC-β for the substrate PIP_2_ by 10-fold caused an expected stimulatory effect, raising intracellular calcium and steady-state levels of cytosolic inositol phosphates (IP_3_, IP_2_, and IP) between 2- and 3-fold. Interestingly, reaction fluxes for phosphoinositide hydrolysis were diminished, possibly due to substrate depletion. Taken together, these examples illustrate the system-wide effects of perturbations in the kinetic rate processes. The procedure could easily be extended to examine multiple simultaneous perturbations in both reaction rates and steady-state concentrations. In future applications of this approach, genomic or proteomic information of multiple perturbations could be used to help predict platelet signaling phenotypes.

### Top-down signaling approaches

**Figure 5:**
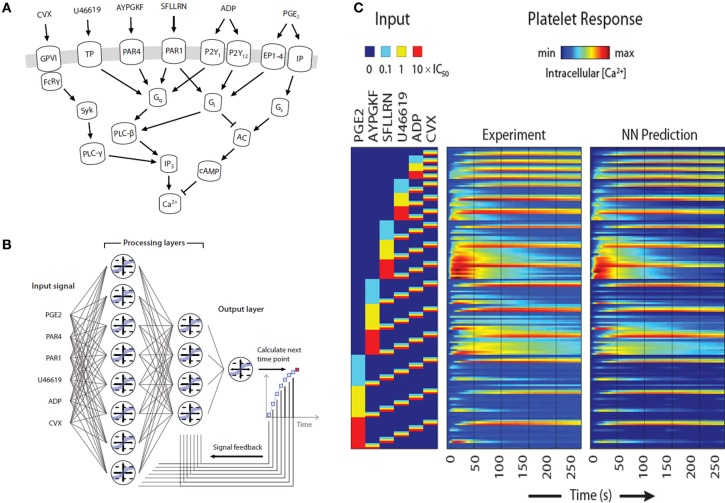
**Pairwise agonist scanning to predict global calcium response in human platelets. (A)** Simplified schematic of signaling pathways examined in this study that converge on intracellular calcium release in human platelets. **(B)** Dynamic NN model used to train platelet response to combinatorial agonist activation. A sequence of input signals representing agonist concentrations is introduced to the network at each time point. Processing layers integrate input values with feedback signals to predict the next time point. **(C)** A total of 154 calcium traces were measured for single and pairwise activation using 6 different agonists (“Experiment”) and used for neural network training. The NN training accurately predicted (“NN Prediction”) the training data.

The prior example required about 200 parameters to describe P2Y1 signaling. In contrast, *top-down* approaches in systems biology allow the construction of large data sets to predict system response without precise knowledge of intracellular pathways. During thrombosis, platelets respond simultaneously to collagen activation of GPVI and α_2_β_1_, ADP activation of P2Y_1_, P2Y_12_, and P2X_1_, thromboxane activation of TP, and thrombin activation of PAR1 and PAR4, while NO and PGI_2_ dampen responsiveness (Figure 5A). We have developed a 384-well plate assay to measure platelet calcium mobilization in response to single and pairwise agonist stimulation at 0.1, 1, and 10XEC_50_, a method termed PAS (Chatterjee et al., 2010). We developed a six agonist probe set for activation of P2Y_1_/P2Y_12_, PAR1, PAR4, TP, IP, and GPVI pathways and measured the EC_50_ for each agonist (ADP, EC_50_ = 1.17 μ M; SFLLRN, 15.2 μ M; AYPGKF, 112 μ M; U46619, 1.2 μ M; PGE_2_, 25 μ M; and convulxin, 0.005 μ M). To capture how the 6 molecular signals are processed by activated platelet, we trained a 2-layer neural network (NN) model (Figure 5B) that predicted time-course behavior of the training set of pairwise combinations of the six agonists (Figure 5C). We used a nonlinear autoregressive network with exogenous inputs (NARX) model to predict successive time points from all 154 Ca^2+^ release curves gathered by experiment. The NN model, which was trained on pairwise agonist stimulation with both agonists added simultaneously, was successful in predicting response to sequential addition of agonists and ternary agonist stimulation (Chatterjee et al., 2010). With 4077 NN simulations fully spanning the 6 dimensional agonist space, 45 combinations of 4, 5, and 6 agonists (predicted to range from strong synergism to strong antagonism) were selected and confirmed experimentally (*R* = 0.88), revealing a highly synergistic condition of high U46619/PGE_2_ ratio, consistent with the known thrombotic risk of COX-2 therapy.

Furthermore, PAS provided 135 pairwise synergy values that allowed a unique phenotypic scoring and differentiation of individuals. We measured synergy vectors for 10 healthy donors in replicate. From clustering analysis, 7 of 10 donors self-clustered when tested twice in a 2-week period, revealing at least two major phenotype classes. Thus, PAS offers a sensitive, patient-specific experimental and computational platform for understanding how a cell integrates many inputs. The trained NN is ideal for use in a multiscale model of clotting under flow conditions.

### Platelet interactions with the vessel wall

**Figure 6:**
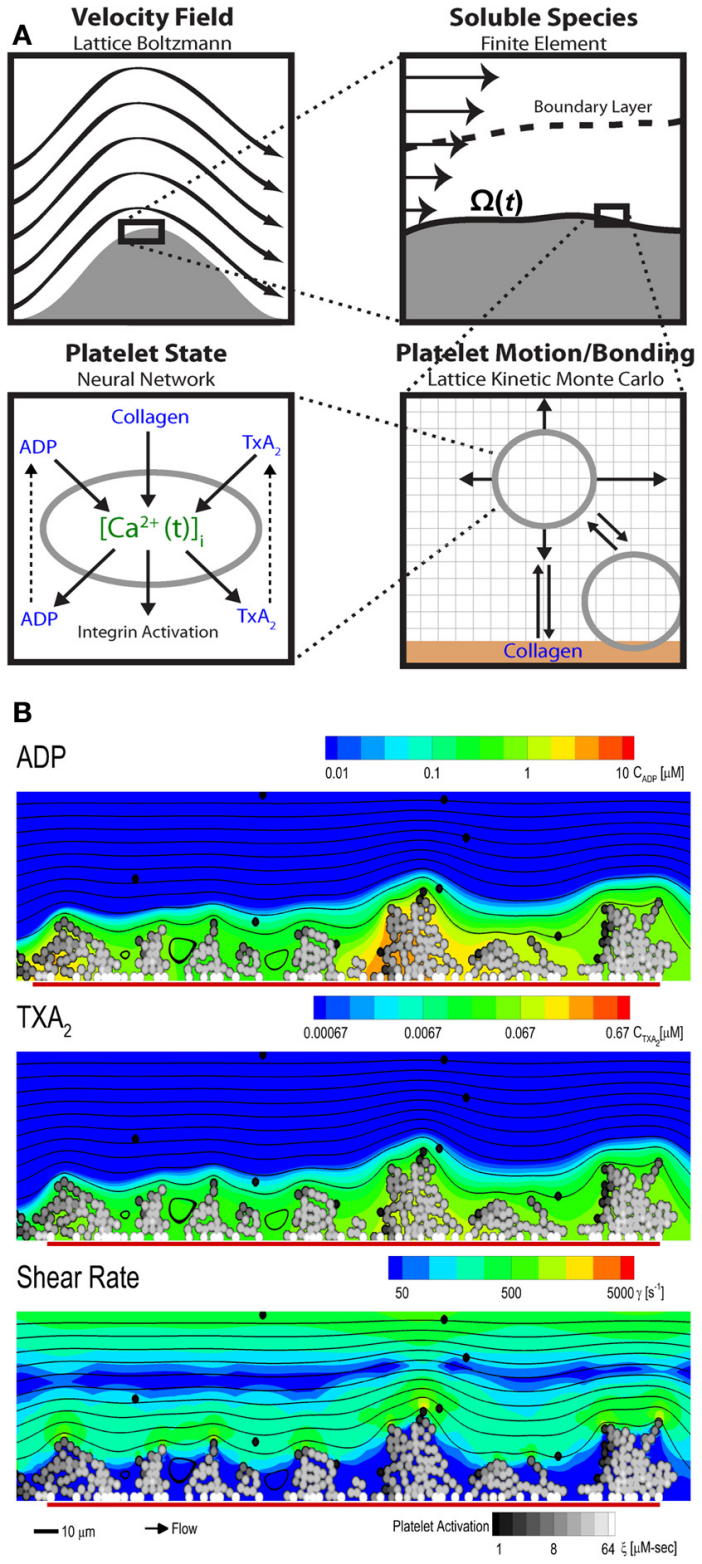
**Multiscale modeling.** The multiscale model has four main components **(A)** fluid flow, transport of soluble species, motion and binding of platelets, and the activation state of each platelet. The fluid flow is perturbed by the growing clot and is determined using the lattice Boltzmann method. The released soluble agonists form a boundary layer in the flow, and this process is determined using the finite element method. Platelet motion and bonding are simulated with lattice kinetic Monte Carlo. Platelet activation state is estimated from the history of intracellular calcium concentration, which is determined by a neural network model. **(B)** Multiscale simulation of patient-specific platelet deposition under flow for a specific donor and PAS-trained neural network of calcium signaling. Platelet activation (black, unactivated; white, activated) and deposition at 500 s (inlet wall shear rate, 200 s^−1^) showing released ADP (top) and TXA_2_ (middle) and perturbation of the flow field (bottom). Flow: left to right (streamlines, black lines); surface collagen (250 μm long): red bar.

The multiscale systems biology model accommodates platelet signaling, platelet adhesion to collagen and other activated platelets, release of soluble agonists, thrombus growth, and distortion of the prevailing flow field (Figure 6A). The lattice Boltzmann (LB) method is used to solve for the velocity field of the fluid. Platelets in the growing aggregate release ADP and TXA2 into the fluid, and a boundary layer is formed with the flow. The dynamics of this process are determined with a finite element method solution of the convection-diffusion-reaction equation for each of the soluble species, ADP and TXA_2_. Platelets move in the fluid by convection and RBC-augmented dispersion. They also bind to the collagen surface as well as previously bound platelets. The motion and binding of platelets is simulated using the convective lattice kinetic Monte Carlo (LKMC) algorithm validated for stochastic convective-diffusive particle transport (Flamm et al., 2009, 2011, 2012). The level of integrin activation and associated adhesiveness for each platelet is related to the cumulative intracellular calcium concentration. The intracellular calcium concentration is determined using a NN trained on a specifc patient's platelet PAS phenotyping experiment. Using this multiscale approach, Multiscale simulations predicted the density of platelets adherent to the surface, platelet activation states, as well as the spatiotemporal dynamics of ADP and TXA_2_ release, morphology of the growing aggregate, and the distribution of shear along the solid-fluid boundary (Figure 6B). Platelets stick to the collagen surface and release ADP and TXA_2_ which forms a boundary layer extending up to 10 μm from the thrombus. Boundary layer concentrations of up to 10 μ M ADP and 0.1 μ M TXA_2_ were found by simulation. TXA_2_ concentrations were found to be sub-physiological (<0.0067 μ M or <0.1 xEC_50_) until a sufficient platelet mass accumulated at the surface after ~250 s. Boundary layer ADP concentrations were within the effective dynamic range (0.1–10 μ M) throughout the simulation. The strong temporal and spatial fluctuations in the concentration of ADP were predominately driven by the short release time (5 s), whereas the longer release time of TXA_2_ (100 s) smoothed fluctuations. The shear rate along the solid-fluid boundary became nonuniform during the simulation (5–10-fold increase above 200 s^−1^) due to surface roughness. At 500 s, the platelet deposit was characterized by platelet clusters 20–30 μm in length, fully consistent with microfluidic measurements of platelet cluster size on collagen at this shear rate (Colace et al., 2011). Platelet accumulation rates on collagen as detected using microfluidic chambers (Maloney et al., 2010) and were highly consistent with simulation predictions for 3 separate donors (each with a trained NN model) in the presence of TXA_2_ antagonism (indomethacin or aspirin), ADP antagonism with a P2Y_1_ inhibitor, or IP activation (with iloprost).

## Conclusion

For multi-scale modeling of platelet-vessel wall interactions, a given modeling route at each scale has advantages and disadvantages. Top-down models (like NNs) are most easily obtained in a patient-specific manner to describe platelet function, however they typically fail to identify specific intracellular regulators. Bottom-up models (like ODE models) of platelet signaling provide molecular precision but face three challenges: (1) difficulty in parameterization, (2) difficulty in fitting to high dimensional data, and (3) incomplete knowledge. Both NN and ODE models are both relatively fast in terms of computational speed. Lattice kinetic Monte Carlo (LKMC) methods provide a balance of speed and sub-micron spatial precision, particularly for discrete cellular systems over millimeter-scale phenomenon and 100 or 1000s of cells. LKMC methods are also relatively easy to program and facilitate the passing of information with other lattice based methods (like Lattice Boltzman or finite elements). LKMC methods become exceedingly slow for molecular simulations of large ensembles when time steps become impossibly small. For solving 2D flow problems, Lattice Boltzman is relatively fast and easy to implement and has no issues of numerical stability. One of the biggest numerical challenges is solving multi-component, reaction-diffusion problems with spatial gradients. Wall-derived TF triggers coagulation and ~10–100 PDEs must be solved to calculate thrombin and fibrin levels in a growing thrombus. Solving large PDE systems is especially computationally intensive (days or weeks) and resists parallel processing.

A central goal in blood systems biology is to elucidate the regulatory complexity of cellular signaling across a large ensemble of interacting cells responding to numerous spatiotemporal stimuli in the presence of pharmacological mediators, ideally in a patient-specific and disease-relevant context (i.e., containing hemodynamics). Developing tools to define platelet variations between patients and the relationship of platelet phenotype to prothrombotic or bleeding traits will have significant impact in stratifying patients according to risk. This multiscale approach also makes feasible patient-specific prediction of platelet deposition and drug response in more complex *in vivo* geometries such as stenosis, aneurysms, stented vessels, valves, bifurcations, or vessel rupture (for prediction of bleeding risks) or in geometries encountered in mechanical biomedical devices.

### Conflict of interest statement

The authors declare that the research was conducted in the absence of any commercial or financial relationships that could be construed as a potential conflict of interest.
